# Supplementary material for: Polysome Profiling Method for Low-Input Human Postmortem Brain
Source: bioRxiv. 2026 May 29:2026.05.28.726378. Preprint. [Version 1] doi: 10.64898/2026.05.28.726378 (PMC13232300; doi:10.64898/2026.05.28.726378)
Supplement: Supplement 1 [file media-1.zip › Sharma_Protocol_Supplementary-Info.pdf]

Supplementary Information

Buffer Formulations and Sucrose Gradient Preparation Details

Table S1: Gradient Buffer (pH 7.4)

Reagent	Final concentration	Amount
Tris-HCl pH 7.4 (1 M)	20 mM	1.4 mL
$MgCl_2$ (1 M)	5 mM	0.35 mL
NaCl (4 M)	150 mM	2.62 mL
Glycerol	8 %	5.6 mL
*DTT (500 mM)	1 mM	0.14 mL
*Cycloheximide (100 mg/ml)	0.1 mg/ml	0.7 mL
DEPC H_2O	n/a	59.19 mL
**Total**		**70 mL**

Table S2: DEPC-treated water

Reagent	Final concentration	Amount
DEPC	0.1 %	1 mL
ddH_2O	n/a	999 mL
**Total**		**1000 mL**

NOTE: DEPC does not dissolve immediately in water; stir (preferably overnight) until the globules disappear. Autoclave to degrade DEPC and allow the solution to cool to room temperature before use.

Table S3: Sucrose Gradient Formulations (10–50%) Prepared from 55% Stock

Sucrose	Amount of 55% sucrose	Amount of gradient buffer
50%	4.54 mL	0.46 mL
40%	3.63 mL	1.37 mL
30%	2.72 mL	2.28 mL
20%	1.81 mL	3.19 mL
10%	0.90 mL	4.10 mL

NOTE: 5 ml of 10-50% sucrose buffers are sufficient to prepare 6 tubes (SW41Ti).

Table S4: Lysis Buffer (pH 7.4)

Reagent	Final concentration	Amount
Tris-HCl (1 M) pH 7.4	20 mM	20 µL
$MgCl_2$ (1 M)	5 mM	5 µL
NaCl (4 M)	150 mM	37.5 µL
Glycerol	8%	80 µL
Triton-X (10%)	1%	100 µL
#Sodium deoxycholate (10%)	1%	100 µL
*DTT (500 mM)	1 mM	2 µL
*Cycloheximide (100 mg/mL)	100 µg/mL	1 µL
*PMSF (200 mM)	1 mM	5 µL
*Protease inhibitor EDTA free (10X)	1 X	100 µL
*Turbo DNAse (2U/µL)	24 U/mL	12 µL
*RNase Inhibitor (40U/µL)	100 U/mL	2.5 µL
DEPC H_2O	n/a	632.5 µL
**Total**		**1 mL**

*# Used only for brain tissue; *Add fresh, just before use*

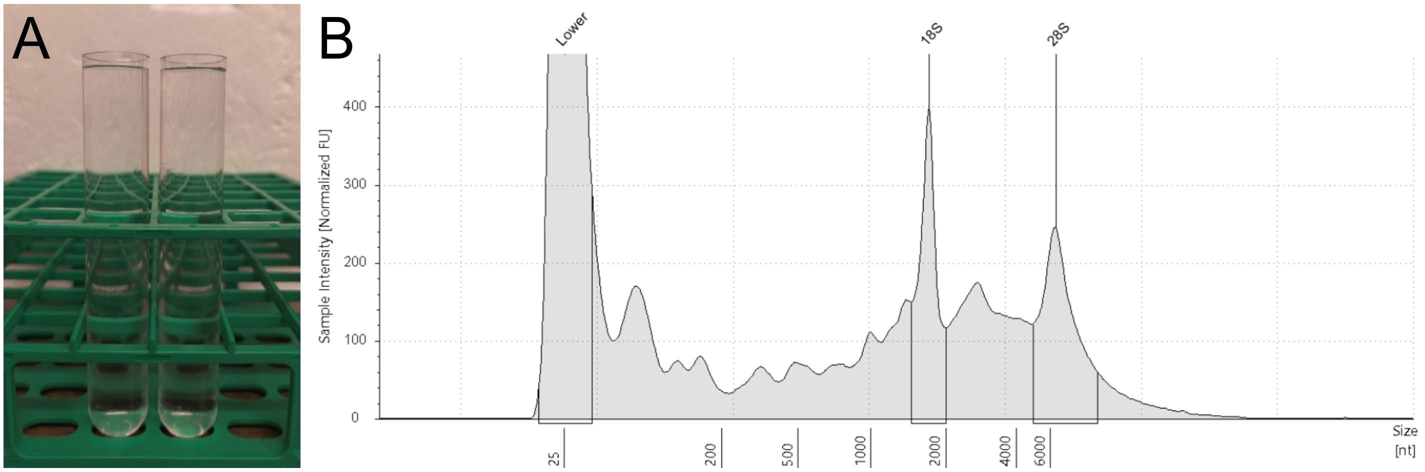

Sample Table

Well	RINe	28S/18S (Area)	Conc. [ng/µl]	Sample Description	Alert	Observations
H8	6.7	1.2	11.1	#185		

Peak Table

Size [nt]	Calibrated Conc. [ng/µl]	Assigned Conc. [ng/µl]	Peak Molarity [nmol/l]	% Integrated Area	Peak Comment	Observations
25	40.0	40.0	4710	-		Lower Marker
1704	1.49	-	2.57	45.30		18S
6383	1.80	-	0.830	54.70		28S

Figure S1. RNA quality control and sample preparation. (A) Ultracentrifuge tubes placed in a snug-fit rack and filled to ~95% of total volume to ensure stability during centrifugation. **(B)** Representative electropherogram displaying RNA size distribution of the human postmortem sample used for polysome profiling.
